# Evaluation of Paediatric Nursing Students' Medication Calculation Competence: An Observational Study Using a Gamified Contest

**DOI:** 10.1002/nop2.70295

**Published:** 2025-09-25

**Authors:** Pablo Buck Sainz‐Rozas, Evelin Balaguer‐López, Pablo García‐Molina, Pedro García Martínez

**Affiliations:** ^1^ Facultad de Enfermería y Podología Universidad de Valencia Valencia Spain; ^2^ Hospital Universitari Vall d'Hebron Barcelona Spain; ^3^ Hospital Clínico Universitario de Valencia Valencia Spain; ^4^ Care Research Group INVESTENF‐INCLIVA Valencia Spain; ^5^ Research Group GREIACC Health Research Institute La Fe Valencia Spain

**Keywords:** drug dosage calculations, nurse education, pharmacology, SafeMedicate, student nurse

## Abstract

**Aim:**

To analyse the general ability of second‐degree nursing students at the ‐REDACTED‐ to calculate, prepare, and administer paediatric medication.

**Design:**

A descriptive, analytical and cross‐sectional study was carried out through a gamified educational innovation project: “Medication Contest” following the STROBE statement.

**Methods:**

The study included all 224 students enrolled in the second year of nursing. The expert faculty created and validated an ad hoc questionnaire with 30 multiple‐choice questions. These responses were analysed to evaluate mathematical, conceptual, and concentration‐related competencies. Secondary variables were calculated, and correlations with the total score were examined. All variables were correlated with the final score, and their predictive capacities were analysed. To differentiate competencies, the 50 highest scores were compared to the rest of the participants.

**Results:**

Out of the 224 participants, 50 qualified for the second phase, with 37 achieving the best results. The median final score was 3/30 (IQR 6.3), differentiating 8.8 and 2.3 (*p* < 0.001) between those included and not in the top 50. Twenty‐three primary variables and all secondary variables significantly correlated with the total score. Mathematical competence was the main predictor variable of the final score (*R* ^2^ = 0.609). All secondary variables were discriminatory.

**Public Contribution:**

The general ability to calculate, prepare, and administer paediatric medication in the 2nd year nursing population is deficient. Mathematical competence is the predictor variable of the final score, identifying an area of intervention and the evaluation on which to intervene.

**Tweetable Abstract:**

Gamification enhances medication calculation training for nursing students through competitive quizzes and simulations.

## 1 Introduction

Medication errors in nursing practice constitute between 50% and 68.1% of errors reported in health systems (Jember et al. 2018; Vaziri et al. 2019; Wondmieneh et al. 2020) and are the main cause of injuries and avoidable damage to them (Donaldson et al. 2017). In Spain, the main professional reporting of these incidents has been nurses, with 64.86% of the total notifications in 2021, and the error associated with the use of medication in the healthcare environment is the second most common type of incident, with 16.4% of notifications (Ministry of Health 2023). Among these errors, calculation errors were the most frequent, which could have serious consequences, such as death, hospitalisation, or disability of users (U.S. Food and Drug Administration 2019).

Medication errors pose a significant risk to patients, being three times more likely to occur among paediatric patients, with rates exceeding 70%. It is nurses who are at the forefront of these errors, with rates between 72% and 79% (Marufu et al. 2022). Medication errors by nursing professionals occur mainly during the administration phase and are associated with an incorrect dose, time, or patient (Latif et al. 2013). Although the causes are multifactorial, lack of knowledge in pharmacology and poor calculation skills have been highlighted as factors that contribute to these errors (Escrivá Gracia et al. 2019; Afaya et al. 2021; Stolic et al. 2022).

The complexity in the management of medications and their relationship with patient safety means that the training level of nurses must be high (Rohde and Domm 2018), especially when the profile of patients is increasingly fragile, with people older and with chronic pathologies and more complex treatments (Gill et al. 2019). International literature has highlighted the lack of preparation and inadequate knowledge among nurses to provide safe care, and questions have been raised about the suitability of existing educational models (Escrivá Gracia et al. 2019).

Medication errors in paediatrics occur more frequently than in adults, posing a major risk to patient safety. Studies show an error rate of 14.8 per 1000 patient‐days in paediatric units compared to 5.7 in adult units, with adverse drug events being three times higher in children (Lan et al. 2014; Kaushal et al. 2001). Factors such as weight‐based dosing, dilution of stock solutions, and immature physiological systems contribute to this risk (Lan et al. 2014). The most common phase for errors is medication preparation and administration, where nurses are responsible for up to 59% of incidents (Ross et al. 2000). Emergency services also show high rates of miscalculations in drug dosing (10%–31%). The primary causes of these errors include poor calculation skills and limited pharmacological knowledge, with frequent issues related to unit conversions and mathematical operations (Wong et al. 2009; Caruso et al. 2015; Samuels‐Kalow and Camargo 2019).

Medication safety use competencies should be reinforced throughout the nursing curriculum (Fusco et al. 2021), beginning as early as possible in the nursing curriculum and maintained throughout the training process. In our context, calculating medication doses is a skill that is acquired transversally throughout the training process and in which different subjects participate: Pharmacology, Child and Adolescent Health Nursing, or different practicums (Ministry of Universities 2023). To achieve this learning, the predominant educational methodology in different subjects continues to be the master class (Ministry of Universities 2023), although other methods such as e‐learning or gamification have shown greater benefits in the student learning process (Lee and Lin 2013; Razaghpoor et al. 2024).

## 2 Background

During the last few decades, innovative strategies have been applied in learning to use medications. These strategies include the use of Kahoot! (Bryant et al. 2018), board games (Quinn 2016; Cutumisu et al. 2019) and YouTube (Zamora et al. 2017). Games could be a valuable medium for higher education (Lickiewicz et al. 2020; Gonzalo‐Iglesia et al. 2018; Taspinar et al. 2016) since their qualities include intrigue, fun, and entertainment. In addition, games increase students' commitment, motivation, cooperation, communication skills, critical thinking, and learning satisfaction (Karbownik et al. 2016; Gonzalo‐Iglesia et al. 2018; Pisano et al., 2020; Taspinar et al. 2016).

Various studies have shown the benefits of gamification or the use of games as learning strategies, improving the management of clinical protocols (Pisano et al., 2020), application of cardiopulmonary resuscitation manoeuvres (Cutumisu et al. 2019; Buck et al. 2022), and study of human physiology (Luchi et al. 2019). Although studies have not shown significant long‐term differences, greater short‐term learning has been observed in groups with gamification (Jones et al. 2015; Karbownik et al. 2016), and students' perceptions have been positive both for retaining knowledge and coping with their exams (Wingo et al. 2019). Recent longitudinal research by Rodrigues et al. (2022) suggests that gamification initially suffers from a novelty effect, where its impact declines after 4 weeks, but it later benefits from a familiarisation effect, recovering its effectiveness between 6 and 10 weeks.

The design of university training strategies aims to increase motivation, strengthen knowledge retention, and improve learning results (Chang et al. 2022). All these objectives can be achieved by applying gamification to learning.

Dutra et al. (2022) highlighted that training in medication calculation for undergraduates must incorporate a guide to internalise the new information (Aydin and Dinç 2017), organise an integrated clinical practice, administer medication, calculate dose as close to real life as possible, and obtain a multidimensional learning experience. This experience should include visual, motor, emotional, and/or tactile resources that improve the learning process and recovery of information through the simulation of practices in the work unit (Dutra et al. 2022).

At the Faculty of Nursing ‐REDACTED‐, an interactive, gamified and competitive learning intervention was planned and implemented: “Medication Contest” following the principles of intelligent teaching (Ambrose et al. 2010) with the aim of improving the learning of medication management in paediatric patients among undergraduate Nursing students. Intelligent learning is based on prior knowledge, student motivation, practical application of knowledge, and self‐monitoring.

The general objective of this study was to analyse the general ability of second‐year undergraduate nursing students to calculate, prepare, and administer paediatric medication.

As secondary objectives, the aim is to analyse the relationship between general calculation capacity, preparation, and administration of paediatric medication with the complexity and competencies evaluated; identify predictor variables of the general capacity for calculation, preparation, and administration of paediatric medication; and identify the questions that have the greatest discriminant power in the general ability to calculate, prepare, and administer paediatric medication.

## 3 Design

This was a descriptive, analytical, and cross‐sectional study. The study observed performance metrics and did not intervene in student activities beyond gamified evaluations. The study population consisted of students enrolled in the Child and Adolescent Health Nursing subject, taught in the second year of the nursing degree at the Faculty of Nursing ‐REDACTED‐. The study was part of an educational innovation project–with ‐REDACTED‐ code ‐REDACTED‐ consisting of a “medication contest” that, following the teaching guide of the subject, the students had to complete in order to be evaluated. To avoid possible bias due to motivation or prior knowledge, students enrolled in higher‐level subjects and those who did not complete the questionnaires were excluded.

**FIGURE 1:**
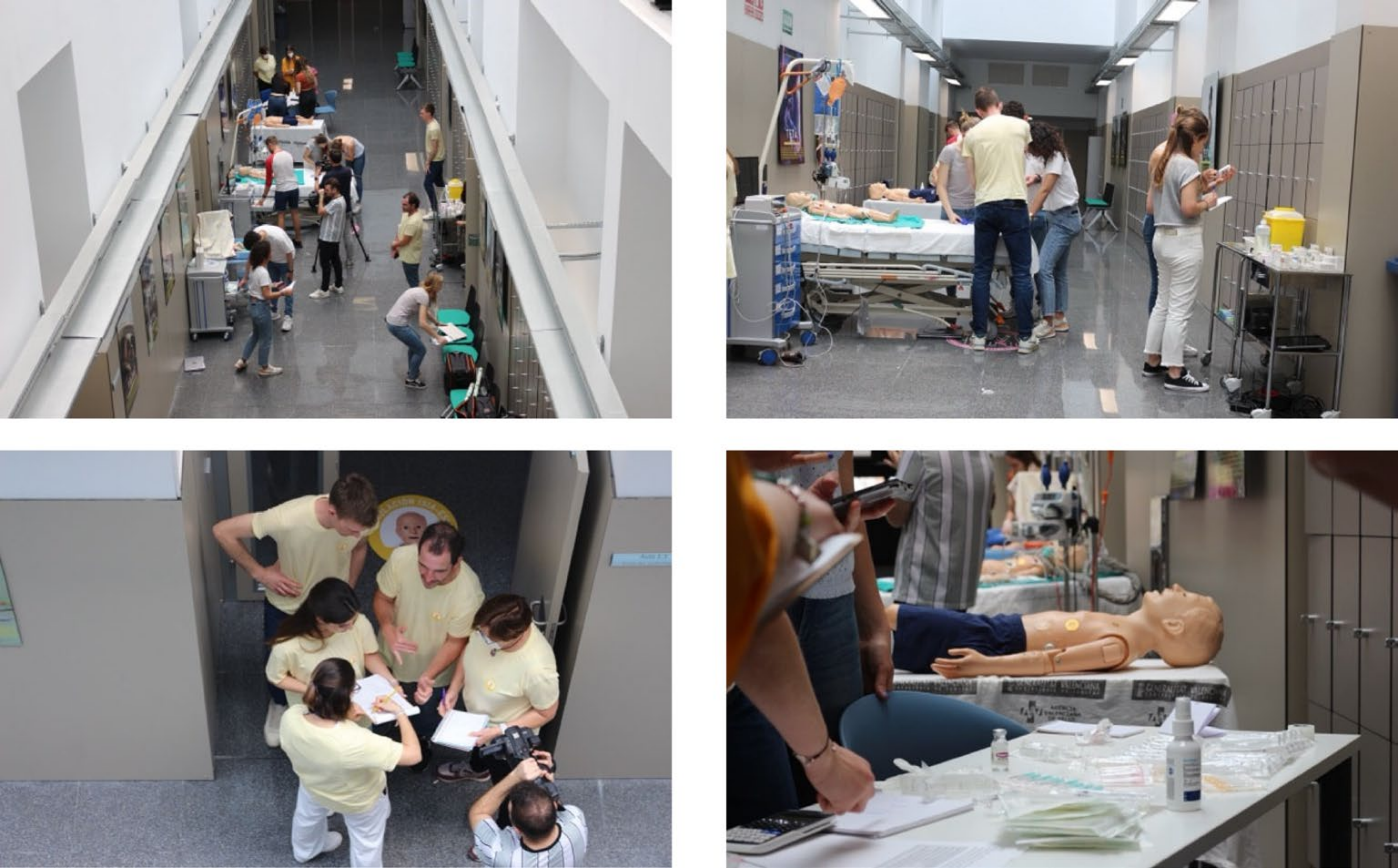
Images 1–4. Implementation of Phase 1 online and Phase 3 of the Medication Contest. Original creation.

The study consisted of four phases:

- Phase 1 included a theoretical session, a practical medication preparation laboratory, and teaching materials, including an Online Escape Room designed to simulate clinical scenarios interactively.
- In Phase 2, an online contest was conducted via a web‐based platform with a 30‐question multiple‐choice questionnaire. The 50 best‐performing students advanced to the next phase. This classification was based on their overall scores.
- Phase 3, the top nine students were selected based on their performance in a subsequent online competition.
- Phase 4, the final in‐person competition, involved advanced clinical simulations. Students performed under timed conditions to assess their medication calculation, preparation, and administration skills. Images 1–4 illustrate the interactive setup, including stations for medication preparation, mock patient administration, and evaluation panels (Figure 1). The STROBE 4.0 statement was followed to maximise the methodological rigour of the study (von Elm et al. 2008).

## 4 Materials and Methods

### 4.1 Instrument and Data Collection

The questionnaire used in Phase 1 was prepared by six teachers of the subjects of Pharmacology, Practicum and Child and Adolescent Health Nursing (CHAN), with more than 20 years of teaching and care experience related to medication calculation. Each teacher developed five multiple‐response questions (four response options) for each condition or competence related to medication errors (Dutra et al. 2022): (a) Mathematical Calculation, (b) Knowledge of Medication Preparation, and (c) Concentration calculation, time. The total number of questions created was 90.

The six teachers classified the questions by competency and complexity levels using a single‐blind technique. After reaching a consensus among the teachers, 30 questions were chosen, as detailed in Appendix 1; Table A1.

To ensure the questionnaire's validity, a face validity assessment was conducted, with faculty members reviewing and refining items based on relevance and clarity. For construct validity, a consensus‐based classification of questions was performed to align them with key competencies in medication calculation. Additionally, reliability testing was conducted using Cronbach's alpha, yielding a coefficient of 0.82, which indicates good internal consistency. Given the absence of an established, validated tool for assessing paediatric medication calculation competence, this study contributes to the development of an instrument adapted to the educational context. Future research should focus on further validation and standardisation of such tools to enhance their applicability.

The questionnaire and sociodemographic variables were entered into Kahoot.it, a web‐based platform where participants completed the test using a unique identifier. The participants were identified using a numerical code whose relationship with the participant was guarded by a professor external to the study.

The data were entered into the IBM SPSS Statistics 28 program, and secondary variables were calculated. Questionnaires that were not completed and records with unregistered usernames were eliminated from the responses, and this elimination was agreed upon by the work team.

### 4.2 Variables

The primary variables were the 30 questions, valued at 1, 0, or −0.33 points indicating success, no response, or error, respectively. This negative marking system was implemented to discourage random guessing and improve the reliability of the assessment, following established methodologies in multiple‐choice testing (Burton 2004; Carneson et al. 1996; Haffejee and Sommerville 2014). As secondary variables, the final score is created as a sum of the points achieved in the 30 items (ranging from 30 to −10 points): easy questions (range from −4.29 to 13), moderate questions (−3.33 to 11), and difficult questions (−1.98 to 6). In addition, mathematical competence (range −4.62 to 14), knowledge competence (range −3.96 to 12), and concentration competence (range −1.32 to 4) are created, all of which are calculated as the sum of the group of questions with this characteristic (Appendix 1; Table A1).

The students who passed Phase 1 (37 2nd year students) were grouped in Group 1 and the rest in Group 2 to identify the questions with the greatest capacity to discriminate the ability to calculate, prepare, and administer children's medication.

### 4.3 Statistic Analysis

The normality of the distribution of the quantitative variables was analysed using the Kolmogorov–Smirnov test, and when a non‐normal distribution was present, they were described by median and interquartile range. The correlation of the secondary variables with general capacity was analysed using Spearman's correlation test, and a predictive model was developed using linear regression of general capacity. An analysis of the comparison of means of the primary variables between Groups 1 and 2 was carried out using the Mann–Whitney *U* test to identify the questions with the greatest discrimination capacity. IBM SPSS Statistics 28 was used for all analyses, and the significance was set at *p* < 0.05.

### 4.4 Ethical Considerations

The study was conducted in accordance with the ethical recommendations for research in the Declaration of Helsinki (AMM 1964) and subsequent updates. The intervention did not imply a risk or a negative impact on the participants. Ethical approval was not applicable, as the intervention was an educational innovation integrated into the course curriculum, following institutional research ethics guidelines and European research integrity standards (ALLEA 2018). Additionally, in line with the Spanish National Declaration on Scientific Integrity (CRUE, COSCE, and CSIC 2015), studies embedded in routine coursework that do not involve additional interventions or sensitive data collection are exempt from formal review. Participants signed a data protection clause and agreed to the terms and conditions of participation.

The data were anonymised and stored on a secure server at the promoter centre as well as at the company Kahoot.it. The treatment, communication, and transfer of personal data of all participating subjects complied with legal regulations according to EU Regulation 2016/679, guaranteeing confidentiality at the level of protection of current legislation in our country.

## 5 Results

**FIGURE 2:**
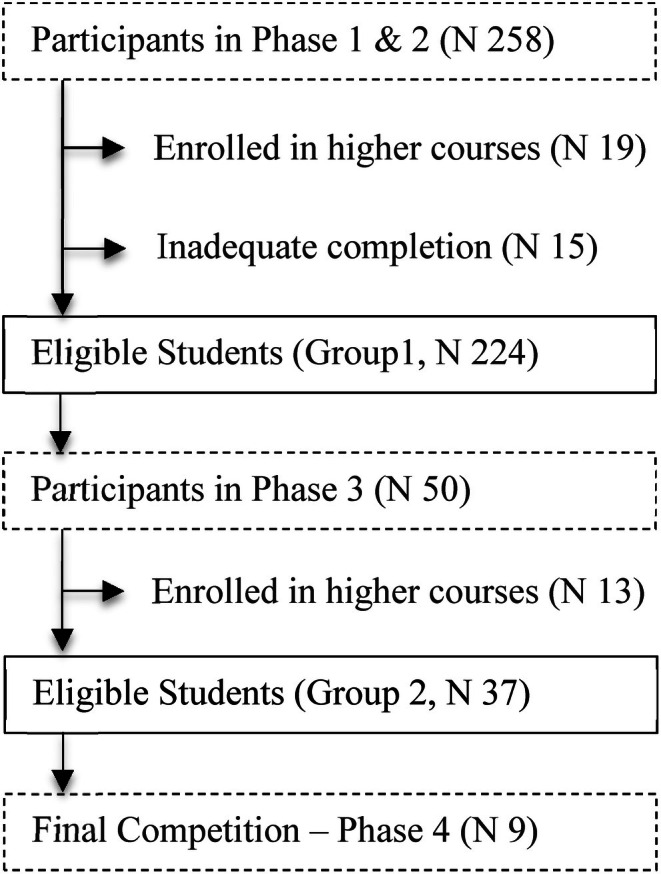
Flow diagram for selecting the study population. *Original creation*.

The study sample comprised 258 students, of whom 88.37% were female (*n* = 228) and 11.63% were male (*n* = 30). The mean age of the participants was 22.08 years (SD = 5.34). Among the participants, 70% had already completed or were currently enrolled in the pharmacology subject. Of the initial 258 participants, 19 were excluded due to enrolment in higher‐level subjects, and 15 did not complete the questionnaire. Of the 50 students who passed Phase 1, 13 were excluded because they were not enrolled in the CHAN subject (See Figure 2). The final participant group included 224 students enrolled in the CHAN subject, who were grouped into Groups 1 (*n* = 37) and 2 (*n* = 187).

All variables, both primary and secondary, had non‐normal distributions. Appendix 1; Table A1 shows the descriptive study of the primary variables, their correlation with general ability, and the comparison of means between Groups 1 and 2. Table 1 shows a descriptive study of the secondary variables and their correlations with general capacity. A high level of significance in the correlation of the primary variables (23 out of 30) and secondary variables (all) with general ability stands out.

**TABLE 1:** Number of questions and score for each category, in the first phase in 2nd year students.

	*N*°	*M*	IQR	*p* ^a^
**Complexity**				
Low	13	1.03	2.66	< 0.001*
Moderate	11	0.68	1.66	< 0.001*
High	6	1.35	3.22	< 0.001*
**Competence**				
Mathematical Calculation	14	1.03	3.03	< 0.001*
Knowledge of Medication Preparation	12	1.02	2.69	< 0.001*
Concentration calculation, time	4	0.01	1.34	< 0.001*
General Capacity	30	3	6.3	

Abbreviations: IQR, interquartile range; *M*, median; *N* ^o^, quantity.

^a^ Spearmans' Rho Test.

* Significance level < 0.001.

When carrying out a stepwise predictive regression model, all secondary variables were introduced, and applying the principle of parsimony, mathematical competence was identified as the predictor variable (*R* ^2^ = 0.609).

The analysis of Groups 1 and 2 shows us a result for the general ability in Group 1 of: median = 8.8 points and IQR = 3.9, and for Group 2: median = 2.3 and IQR = 2. The comparison of means identifies significant differences in 13 primary variables (Appendix 1; Table A1) and all secondary variables (Table 2).

**TABLE 2:** Comparison of scores for each category between students who pass (Group 1) or do not pass (Group 2) to the second phase.

	Group 1		Group 2		*p* ^a^
	*M*	IQR	*M*	IQR	
**Complexity**					
Low	4.02	3	0.69	2.02	< 0.001**
Moderate	3.35	1.66	0.69	2.35	< 0.001**
High	0.68	2.17	0.01	1.33	0.001*
**Competence**					
Mathematical Calculation	4.02	3	1.02	2.66	< 0.001**
Knowledge of Medication Preparation	3.02	3.99	0.36	2.66	< 0.001**
Concentration calculation, time	1.34	1.33	0.01	1.67	< 0.001**
General Capacity	8.8	3.9	2.3	4.2	< 0.001**

Abbreviations: IQR, interquartile range; *M*, median.

^*a*^ Mann–Whitney *U* test.

* Significance level < 0.005.

** Significance level < 0.001.

The 13 primary variables with significant differences between Groups 1 and 2 (Appendix 1; Table A1) were distributed by competencies in six (46.15%) mathematics, five (38.46%) knowledge and two (15.4%) concentration areas. By complexity, the distribution was: easy 6 (46.15%), moderate 6 (46.15%) and difficult 1 (7.7%). The final structure of these 13 variables maintained the distribution in percentage of competence of the original questionnaire, but they modified the representation of the difficult questions, which decreased from 20% to 7.7%, and the weight of the moderate questions increased from 36.67% to 46.15%.

## 6 Discussion

The general ability to calculate, prepare, and administer paediatric medication was low, with 99.11% of participants not passing the evaluation questionnaire (score less than 15 out of 30 points). These results are in line with those presented by other authors who highlight the lack of knowledge in pharmacology and poor skills in calculating pharmacological doses (Escrivá Gracia et al. 2019; Afaya et al. 2021; Stolic et al. 2022). Furthermore, it reinforces the need to begin as soon as possible to develop competence related to pharmacological administration in the nursing curriculum and maintain it throughout the training process (Fusco et al. 2021); it also creates a multidimensional learning experience that includes visual, motor, emotional, and/or tactile resources, such as the use of medication containers, vials, syringes, or simulated environments that improve the learning process and the recovery of information through the simulation of practices in the work unit (Dutra et al. 2022).

The CHAN course incorporates various active learning strategies, each tailored to specific objectives. While simulation and problem‐based learning enhance clinical reasoning, gamification is particularly effective for medication calculations, promoting engagement and reinforcing key concepts interactively. Evidence from Dutra et al. (2022) and Gill et al. (2019) supports strategies that build on prior knowledge and structured tasks, aligning gamification as an optimal approach for achieving course goals.

The creation of innovative projects through gamification has already shown benefits (Karbownik et al. 2016; Gonzalo‐Iglesia et al. 2018; Pisano et al., 2020; Taspinar et al. 2016) and can improve this situation, and this is how they have been developed in the Faculty of Nursing ‐REDACTED‐ (‐REDACTED‐) with great acceptance by students and professionals.

The predictive model showed that mathematical competence had a predictive capacity of 60.9% of the general competence of the students. This data is worrying since, although it is true that mathematical calculation is transversal knowledge in nursing, it is understood that basic competencies in mathematics are developed in primary and secondary education (Ministry of Education 2022). However, solving these mathematical problems using the ‘rule of three,’ a proportional reasoning method, can create a disconnect for students. This approach, traditionally favoured by previous generations (Beltrán‐Pellicer and Alsina 2022), is currently being reconsidered under new educational laws (Ministry of Education 2022).

This intergenerational conceptual complexity does not seem to be the only cause for the limitations of mathematical competence. The PISA 2018 report (Ministry of Education 2019) indicates that the mathematics competence of 15‐year‐old Spanish students is slightly lower than that of the European Union (481 vs. 494), and that of the Valencian Community (473 points) is clearly lower, being in any case at level 2 of mathematical competence of the six proposed. These results have been maintained in the PISA 2022 report (Ministry of Education 2023) (478 Spain, 475 Valencian Community). Therefore, a solution is not foreseen from pre‐university educational levels to this competition, which may have a negative result in the security of the patient.

The comparative study between Groups 1 and 2 showed a very high difference in scores (greater than 6 in the median) between both groups. Despite this, only two participants exceeded 15 points on the questionnaire and passed a standard exam. Among the 13 primary variables that made the difference between both groups, a difference was observed in the complexity of the questions: the questions with the greatest influence were not the difficult ones but rather the moderate ones. This situation has been classically described (Ebel 1977; García‐Cueto 2005), although it has been questioned by other authors who advocate the presence of questions of the entire range of complexity (Tristán 2006).

It should be noted that the level of complexity of this study was determined by the teaching team that described the questions and not so much by their results in Phase 1 of the contest, so logical differences could be found with this classification in Group 1 (higher score in less difficult questions) that are not observed in Group 2 (equal score in easy and moderate questions), which would call into question a purely numerical classification. This need for the involvement of professionals in the writing and control of the questions used to achieve adequate validity demonstrates the complexity and multifactorial nature of competence in medication calculation, which has already been defended by authors such as Özyazıcıoğlu et al. (2018).

The findings of this study align with global nursing education competencies, such as those outlined by QSEN (Cronenwett et al. 2007) and the WHO patient safety frameworks (Donaldson et al. 2017), which emphasise the importance of medication safety, clinical decision‐making, and error prevention. By incorporating gamification as an active learning strategy, this approach supports the development of critical thinking and engagement, key elements in ensuring safe nursing practice. Future research should further explore how gamification can be integrated into nursing curricula to enhance competency‐based education and contribute to international standards for patient safety and quality of care.

### 6.1 Limitations

The main limitation on the internal validity of this study was the use of a non‐validated instrument for the assessment of competence in medication calculation. An “ad hoc” tool was created because a valid tool for this purpose was not identified. The creation of validated instruments to carry out a more precise assessment of the general ability to calculate, prepare, and administer paediatric medications in nursing is proposed as a future line of research. These tools could be helpful in the selection of personnel in environments that are especially sensitive to patient safety, such as paediatric environments.

Carrying out an ecological study without discriminating variables such as gender, age, academic grades, previous studies, time dedicated to completing the questionnaire, or participation in the activities included in Phase 1 of the project could lead to the non‐identification of confounding factors in the result. Although the research team did not believe that these variables could have an impact on the results, we believe that it is a limitation that should always be assessed. An effort was made to control some confounding variables by considering participants' mean age, excluding higher‐year students, and reporting pharmacology course enrolment. However, prior gamification experience, professional background, and undergraduate performance in logic and calculation were not assessed. In the future, it would be interesting to design a methodology that would allow the measurement of these variables.

Additionally, another limitation of the study is the lack of assessment of the long‐term effects of gamification on knowledge retention. While our findings support gamification as an effective learning strategy in the short term, further research should incorporate a follow‐up study to determine how well students retain knowledge after several months. Understanding the persistence of gamification's impact would provide more comprehensive insights into its true educational benefits.

### 6.2 Applicability

Currently, we have not identified an instrument that adapts to the general ability to calculate, prepare, and administer paediatric medication; therefore, further research is necessary to achieve this. The observational nature of this study allowed us to identify patterns and challenges in nursing students' competencies in a real educational context, providing valuable insights into areas requiring improvement without altering the natural dynamics of the learning process. However, the scalability of this approach to other institutions may be influenced by factors such as curricular differences, available technological resources, and faculty training in gamified methodologies.

The contributions of this study will help teachers, human resource managers, and future researchers to consider mathematical competence as a cornerstone in the acquisition of the general ability to calculate, prepare, and administer paediatric medication. Ensuring the successful implementation of gamification in different academic environments may require institutional support and adaptation to specific learning objectives.

University professors must develop and implement instruments that allow them to diagnose their students' mathematical competence, before developing more complex ones. In addition, these tools would allow for the assessment of the effectiveness of educational interventions that could be implemented and comparisons between populations or over time.

Finally, given the effectiveness of educational innovation strategies based on gamification in developing skills, it is necessary to design studies that assess their effectiveness in the short, medium, and long term. Additionally, future research should explore strategies to overcome potential barriers to implementation, such as resistance to change, the need for continuous methodological adaptation, and integration within competency‐based curricula.

## 7 Conclusion

The general ability to calculate, prepare, and administer paediatric medication in the 2nd year nursing population is poor, and only two of the 224 participants would pass an exam at the level required by university studies.

The general ability to calculate, prepare, and administer paediatric medication is significantly related to all secondary variables, but the secondary variable of mathematical competence has the greatest predictive capacity with respect to the general ability to calculate, prepare, and administer medication. paediatric medication. Finally, 13 of the 30 variables with discrimination capacity were identified in the transition to Phase 2, of which the greatest discriminant capacity of the primary variables of moderate complexity and low discriminant power of the primary variables of high complexity stands out.

In short, the worrying results presented in the field of nursing competence in medication calculation highlight an area in which it is urgent to evaluate and intervene, given the relationship between medication errors and their influence on patient safety.

## Author Contributions

**Pablo Buck Sainz‐Rozas:** conceptualisation, data curation, investigation, project administration, writing – original draft. **Evelin Balaguer‐López:** methodology, software, supervision, validation, writing – review and editing. **Pablo García‐Molina:** conceptualisation, funding acquisition, investigation, resources, writing – review and editing. **Pedro García Martínez:** data curation, formal analysys, methodology, visualisation, writing – original draft.

## Ethics Statement

The study was conducted in accordance with the ethical recommendations for research in the Declaration of Helsinki (AMM 1964) and subsequent updates. The intervention did not imply a risk or a negative impact on the participants. An express description of the use of academic data for research and teaching purposes is found in the teaching guide—accepted by the students—of the subject. The data were anonymised and stored on a secure server at the promoter centre as well as at the company Kahoot. The treatment, communication, and transfer of personal data of all participating subjects complied with legal regulations according to EU Regulation 2016/679 (European Union 2016), guaranteeing confidentiality at the level of protection of current legislation. Our country.

## Conflicts of Interest

The authors declare no conflicts of interest.

## Data Availability

The data that support the findings of this study are available from the corresponding author upon reasonable request.

## Appendix 1

**TABLE A1:** Descriptive study of the questionnaire and complexity and knowledge variables. Correlation of the score of each question with the final score of the questionnaire. Descriptive of the students who pass (Group 1) or not (Group 2) to the second phase and comparative study.

*N*°	Variables	D/C	Me/IQR	*p* ^a^	Group 1 *N* = 37	Group 2 *N* = 187	*p* ^b^
					Me/IQR	Me/IQR	
1	Clara, weighing 10 kg, was prescribed Diazepam at 0.3 mg/kg in 3′–5′ and repeat if necessary every 5′–10′. How is it prepared? (60s): 8 mL of saline are added to the 10 mg/2 mL vial and we administer 3 mL/dose	2/C	−0.33/1.33	< 0.001**	1/1.33	−0.33/1.33	0.009*
2	The Paediatric Intermediate Care Unit has Ketamine in a concentration of 50 mg/mL. Indicate the correct one (60s): For a concentration of 10 mg/mL we take 2 mL of Ketamine and 10 mL of saline.	1/C	−0.33/0	0.018*	−0.33/1.33	−0.33/0	0.083
3	It has this vial containing 10 mL of insulin. How would you prepare a 50 IU dose to a safe concentration of 0.5 IU/mL? (60s): I would take 0.5 mL of the insulin vial dissolved with 100 mL of 0.9% saline.	2/C	−0.33/0.33	0.040*	−0.33/1.33	−0.33/0	0.366
4	3 mcg/kg/min of Nitroprusside is prescribed for a 60 kg man. If I dissolve this ampoule in 250 mL of SG5%… (60 s): The final concentration is 0.2 mg/mL and the infusion rate is 0.9 mL/min	3/C	−0.33/0	0.06	−0.33/1.33	−0.33/0	0.075
5	The safe dose of Aminophylline is 0.36 mg/kg/h and is diluted in 100 mg/100 mL. 59 kg Pepe receives 20 mL/h. (60s): Pepe is receiving a safe dose of 20 mg/h	2/M	−0.33/1.33	< 0.001**	1/1.33	1/1.33	0.981
6	The Midazolam regimen is 0.15 mg/kg at 5 min. Which preparation would be the most correct for a 2400 g neonate? (60s): If the vial is 15 mg/3 mL, take 1 mL and saline up to 10 mL. Administer 0.72 mL.	2/M	−0.33/0	0.296	−0.33/0	−0.33/0	0.544
7	Mioflex vials containing 100 mg/2 mL are available in a Paediatric ICU. Point‐out incorrect dilution. (60s): To obtain 2 mg/mL we take 0.5 mL from the vial and add saline up to 10 mL.	3/M	−0.33/1.33	0.005*	−0.33/1.33	−0.33/0.33	0.522
8	500 mg of IV Vancomycin every 6 h are prescribed. The patient weighs 54.5 kg. The safe dose is up to 40 mg/kg/day (60s): The dose that the patient receives is 36.7 mg/kg/day	2/C	−0.33/1.33	< 0.001**	1/1.33	−0.33/0.33	< 0.001**
9	Oxytocin is dissolved in 5 IU in 500 mL of 0.9% saline. Therefore, 0.5 mL of the ampoule is taken to prepare 1 L of solution (30s): False	1/M	−0.33/1.33	0.001*	1/1	0/1.33	0.014*
10	To prepare an IV infusion of Norepinephrine, 4 mg of Nora is added to 1 L of 5% SG. What is the concentration? (30s): 4 μg/mL	1/M	−0.33/0.33	0.012*	−0.33/0.83	−0.33/0.33	0.232
11	IV amoxicillin 500 mg/6 h is prescribed. We have amoxicillin 1 g/4 mL. How many mL will we administer in 24 h? (30s): 8 mL	1/M	−0.33/1.33	< 0.001**	0/1.33	−0.33/0.33	0.002*
12	You must administer 10 mg of a drug that is available in 5% ampoules. What volume of drug should be administered? (30s): 0.2 mL	1/C	−0.33/0.33	0.050	−0.33/0.83	−0.33/0.33	0.919
13	One 500 mg vial was reconstituted with 10 mL of API. The dose is 125 mg. Take 2.5 mL and dilute it to 50 mL with saline. (60s): The final concentration of your dilution will be 2.5 mg/mL	2/C	−0.33/1.33	< 0.001**	1/1.33	−0.33/1.33	0.002*
14	The IV infusion of a drug was at a rate of 1.5 mL/h. Knowing that it is at a concentration of 500 mg/100 mL (30s): The prescribed dose is 7.5 mg/h	1/M	−0.33/1.33	0.109	0/1.33	0/1.33	0.180
15	You must prepare a solution with this heparin (25,000 IU/5 mL) at a concentration of 100 IU/mL. What would be the total volume of the dilution? (30s): 250 mL	1/C	−0.33/1.33	< 0.001**	1/1.33	−0.33/1.33	0.010*
16	You have this vial of Propofol (200 mg/20 mL) and you have to administer it at a concentration of 5 mg/mL, indicate the incorrect one (60s): You will take 5 ml of Propofol and add 10 mL of physiological saline	1/C	−0.33/1.33	< 0.001**	−0.33/1.33	−0.33/1.33	0.152
17	There are ampoules of Mioflex (Suxamethonium Chloride) of 100 mg/2 mL and 500 mg/10 mL. Indicate the incorrect one (60 s): To get 10 mg/mL take 5 mL of Mioflex 500 mg/10 mL and add saline up to 20 mL	3/T	−0.33/1.33	0.041*	−0.33/1.33	−0.33/1.33	0.200
18	Gentamicin (80 mg/2 mL) is administered at 7.5 mg/kg/day in 1–2 doses. Max concentration 10 mg/mL. Indicate the incorrect one for 9 m and 9 kg (60s): To administer it you must add at least 5 mL to the amount extracted from the vial.	3/T	−0.33/0.33	0.190	−0.33/1.33	−0.33/0	0.113
19	Ana, 7 years old, needs Fentanest, she has 3 mL ampoules of 50 mcg/mL. Indicate the incorrect one (60 s): For a concentration of 0.1 mg/mL you must take 2 mL of Fenta and saline up to 10 mL	2/T	−0.33/0.33	0.013*	−0.33/1.33	−0.33/0.33	0.040*
20	Mr. García was prescribed furosemide (20 mg/2 mL) 70 mg IV along with 40 mL of saline 0.9%. How much furosemide do you need to add? (30s): 7 mL	1/M	−0.33/1.33	< 0.001**	1/1.17	0/1.33	< 0.001**
21	450 mL of 5% Glucose are prescribed for 5 h with a device that administered 20 gts/mL. What is the infusion rate? (30s): 30 drops/min	2/M	−0.33/0.33	0.134	−0.33/1.33	−0.33/0.33	0.078
22	Furosemide should be administered at 2 mg/min IV continuously. The pharmacy sends a bag of Furo 400 mg diluted in 250 mL of D5W. (60s): 75 mL/h will be the infusion rate per hour to be set on the pump	3/M	−0.33/1.33	0.012*	−0.33/1.33	0/1.33	0.888
23	350 mg of IV Gentamicin is prescribed. How many mL of the Genta pictured (120 mg/Vial) should you add to 50 mL of Saline 0.9%? (30s): 5.83 mL	1/M	−0.33/1.33	< 0.001**	1/1.33	−0.33/0.33	< 0.001**
24	1.4 g of IV Benzylpenicillin is prescribed, available in a 650 mg vial reconstituted with 10 mL of API. Indicate the correct one (30s): The final concentration will be 65 mg/mL	1/M	−0.33/0.33	0.008*	−0.33/1.33	−0.33/0.33	0.002*
25	An oral opioid is prescribed at 0.36 g ×4 times/day, it is available in 180 mg tablets. How much will you give in 12 h? (30s): 4 tablets	1/M	−0.33/1.08	0.463	−0.33/1.33	−0.33/0.33	0.368
26	You have adrenaline in solution at a concentration of 10:100,000, what is the concentration in mg/mL? (30s): 0.1 mg/mL	2/T	−0.33/1.33	0.001*	1/1.33	0/1.33	0.015*
27	You have rifampin in 2% suspension. The prescribed dose is 50 mg/24 h. How many mL should be administered per day? (30s): 2.5 mL	2/C	−0.33/1.33	0.001*	1/1.33	0/1.33	0.069
28	It is prescribed to add 2 g of NaCl to a 500 mL saline solution. We have 20% ampoules. How much mL will we add? (30s): 10 mL	3/C	−0.33/1.33	0.027*	1/1.33	−0.33/0.33	0.004*
29	A child (15 kg) is prescribed 20 mg/kg of oral antibiotic in a 150 mg/mL bottle and administered in drops (25 gts = 1 mL) (30s): 50 drops	2/M	−0.33/1.33	< 0.001**	1/1.33	0/1.33	0.007*
30	How many grams of dextrose are needed to prepare 2 L of a 5% solution? (30s): 100 g	1/C	−0.33/1.33	0.007*	0/1.33	0/1.33	0.212

Abbreviations: C, knowledge of medication preparation; IQR, interquartile range; Me, median; *N* ^o^, question number D complexity [1 (low), 2 (moderate), 3 (high)] C, competence [M mathematical calculation); *T*, concentration calculation, time.

^a^ Spearmans' Rho Test.

^b^ Mann–Whitney *U* test.

* Significance level < 0.05.

** Significance level < 0.001.
